# Developing Performance-Based Mix Design Framework Using Asphalt Mixture Performance Tester and Mechanistic Models

**DOI:** 10.3390/polym15071692

**Published:** 2023-03-29

**Authors:** Jong-Sub Lee, Sang-Yum Lee, Tri Ho Minh Le

**Affiliations:** 1 Pavement R&D Office, Korea Expressway Corporation Research Institute, Dongbu-daro 922, Dongtan-myeon, Hwaseong-si 18489, Republic of Korea; 2 Department of Civil Engineering, Induk University, 12 Choansan-ro, Nowon-gu, Seoul 01878, Republic of Korea; 3 Faculty of Civil Engineering, Nguyen Tat Thanh University, 300A Nguyen Tat Thanh Street, District 4, Ho Chi Minh City 70000, Vietnam; lhmtri@ntt.edu.vn

**Keywords:** performance-based, asphalt mix design, performance-related specification, viscoelastic continuum damage model, viscoplastic shift model

## Abstract

This paper proposes a performance-based mix design (PBMD) framework to support performance-related specifications (PRS) needed to establish relationships between acceptable quality characteristics (AQCs) and predicted performance, as well as to develop fatigue-preferred, rutting-preferred, and performance-balanced mix designs. The framework includes defining performance tests and threshold values, developing asphalt mix designs, identifying available performance levels, conducting sensitivity analysis, establishing the relationships between AQCs and predicted performance, and determining performance targets and AQC values for the three PBMDs using predicted performance criteria. Additionally, the framework recommends selecting the PBMD category for each asphalt layer to minimize pavement distresses. In this study, the proposed PBMD protocol was applied to FHWA accelerated loading facility (ALF) materials using asphalt mixture performance tester (AMPT) equipment coupled with mechanistic models. The study developed nine mix designs with varying design VMAs and air voids using the Bailey method. The cracking and rutting performance of the mix designs were determined by direct tension cyclic (DTC) fatigue testing, triaxial stress sweep (TSS) testing, and viscoelastic continuum damage (S-VECD) and viscoplastic shift models for temperature and stress effects. The study found that adjusting the design VMA was the primary way to achieve required performance targets. For fatigue-preferred mix design, the recommended targets were a cracking area of 0 to 1.9%, a rut depth of 10 mm, and a design VMA of 14.6 to 17.6%. For rutting-preferred mix design, the recommended targets were a cracking area of 18%, a rut depth of 0 to 3.8 mm, and a design VMA of 10.1 to 13.1%. For performance-balanced mix design, the recommended targets were a cracking area of 8.1 to 10.7%, a rut depth of 4.6 to 6.4 mm, and a design VMA of 12.6 to 14.3%. Finally, pavement simulation results verified that the proposed PBMD pavement design with fatigue-preferred mix in the bottom layer, performance-balanced mix in the intermediate layer, and rutting-preferred mix in the surface mix could minimize bottom-up cracking propagation without exceeding the proposed rutting performance criterion for long-life.

## 1. Introduction

The Moving Ahead for Progress in the 21st Century Act (MAP-21) transportation bill emphasizes performance and new innovations and technologies for the transportation system’s growth and development [1,2,3]. The Superpave^®^ mix design system was developed under the strategic highway research program (SHRP). However, there has been a need for performance tests to ensure the satisfactory performance of the asphalt mixtures under in-service conditions [4,5,6,7,8]. A performance-based mix design (PBMD) determines the optimal proportions based on predicted performance, which balances competing demands for cracking and permanent deformation [9,10] because these two distresses are somewhat oppositely driven by the relative quantities of aggregate and asphalt binder [11,12,13]. This study suggests a PBMD framework that can support performance-related specification (PRS) required for the mathematic relationships between volumetric asphalt mix designs and predicted performance and develop a fatigue-preferred, rutting-preferred, performance-balanced mix designs that can reduce the critical structural distresses for long-life [7,11,13,14].

The PRS is a specification that describes the desired levels of key materials and construction quality characteristics that have been found to correlate with fundamental engineering properties that predict performance [15]. In order to support a PRS system, a PBMD framework can provide the mathematical models that explain the relationship between the acceptance quality characteristics (AQCs) and predicted performance for selected mixtures [2,11,12,16]. The volumetric and/or mechanical AQCs that can be measured during construction are also identified through the PBMD framework [1,7,16,17,18,19]. Here, the volumetric AQCs are asphalt mix design requirements (i.e., design air void, binder content, design voids in mineral aggregate (VMA)) measured by volumetric tests, whereas mechanical AQCs are simplified performance indices measured by a mechanical performance tester at the time of construction [3,20]. Through the mathematical models, the role of PBMD in PRS is to provide asphalt mix designers guidance on how to adjust asphalt material and volumetric designs to obtain the required performance targets [7,14,16].

Several state departments of transportation (DOTs) have modified current volumetric asphalt mix designs to better control performance-based on mechanical tests (TRB Circular, 2014). In California, polymer-modified binder in the surface layer and rich asphalt bottom layers were constructed for long-life rehabilitation of the I-710 highway [4,18,21]. Polymer-modified binder mixtures are chosen to improve both rutting and fatigue cracking (one might consider this a performance-balanced mix design), whereas the rich binder mixture is focused on better compaction and more resistance to bottom-up cracking and moisture susceptibility (one might consider this a fatigue-preferred mix design) [22]. Furthermore, New Jersey DOT developed structurally oriented asphalt mix designs to prevent critical pavement distresses [15,17,23,24,25]: (1) high performance thin overlay mix design using polymer-modified PG 76-22 binder for rutting performance (one might consider this a rutting-preferred mix design), (2) binder-rich intermediate course mix design using at least PG-70-28 with more binder for reflective cracking performance (fatigue-preferred mix design), (3) bridge deck waterproofing surface course mix design using polymer-modified asphalt binder or concentrated thermoplastic-polymeric asphalt modifier for rut and fatigue performances (performance-balanced mix design), and (4) bottom-rich base course mix design using PG 76-28 binder with a minimum of 5% asphalt binder by weight for bottom-up cracking performance (fatigue-preferred mix design). Most DOTs have already recognized the importance of developing performance-based asphalt mix design systems that can address structural distresses for long-life pavements [15,17,23,24,25].

In pavement engineering, accurate and reliable pavement performance models are crucial for the design and maintenance of roads. However, the calibration and improvement of these models require comprehensive field performance data that are often neglected. Despite the presence of some studies, the importance of in-situ testing and analysis of pavement cracking and rutting performance has not been adequately recognized. Hence, there is a need to emphasize the significance of field performance data in developing and refining pavement performance models. In this regard, numerous studies have highlighted the importance of real-scale measures and performance analysis, as evidenced by recent research [26,27,28].

The concept of a perpetual pavement aims to practice performance-based mix design (and structural design) that can avert bottom-up cracking propagation so that the periodic surface milling and resurfacing can maintain the overall thick pavement over 50 years [29]. A typical perpetual pavement design might consist the following asphalt layers: (1) a surface layer of 40 to 80 mm thickness for rutting resistance, friction, and permeability (possibly a rutting-preferred mix design), (2) an intermediate layer of 100 to 180 mm thickness for rutting and fatigue cracking resistances (performance-balanced mix design), and (3) a bottom layer of 75 to 100 mm thickness for bottom-up cracking resistance (fatigue-preferred mix design).

In this study, the PBMD framework is developed using asphalt mixture performance tester (AMPT) coupled with mechanistic simplified viscoelastic continuum damage (S-VECD) and viscoplastic shift models. Furthermore, asphalt pavement structural analysis program, layered viscoelastic pavement analysis for critical distress (LVECD) developed at North Carolina State University, was used to model fatigue cracking propagation and permanent deformation.

The objective of this paper is to develop the performance-based mix design framework that can provide guidance on how to adjust asphalt materials and designs to achieve required performance targets based on mechanistically predicted performance results that support performance-related specifications for longer life.

## 2. Methods

### 2.1. Current Asphalt Mix Design Methods and Their Requirements

The Hveem mix design method was developed to select the highest asphalt content without exceeding the minimum stability. Hveem evaluates the performance of asphalt mixture by Hveem stabilometer and cohesiometer to measure a resistance to deformation and ravelling. The compacted specimen is fabricated using kneading compactor [30]. Furthermore, the Marshall mix design method was introduced to find optimum asphalt content and density ultimately realized under design traffic. The test specimens are compacted using drop hammer (FHWA, 1988) and the density and optimum binder content were determined by satisfying the criteria of stability and flow tests. Recently, the Superpave^®^ mix design method was developed to increase requirements in aggregate and asphalt binder selection process in addition to the volumetric criteria. Furthermore, it tries to provide rational mix designs applicable for various traffic volumes, axle loads, and climates. Performance tests and models were developed to predict rutting, fatigue cracking, and thermal cracking performance, but were not ready to be implemented at the time. A newer gyratory compaction method was provided because it was found that it orients aggregate particles in the same manner which occurs in the field (Brown, 1989).

The volumetric requirements of the design VMA, and voids filled with asphalt (VFA), and air void have been utilized to control the quality of asphalt mixtures for asphalt mix design for over 25 years. Here, the design VMA is affected by aggregate gradation, aggregate surface texture, aggregate shape, use of manufactured sand, fines and dust content, and so forth. A comparison and contrast of the aggregate and binder selection method, compaction method, and volumetric requirements of the three asphalt mix designs is shown in Table 1.

### 2.2. Development of Performance-Based Mix Design Framework

#### 2.2.1. Performance-Based Mix Design to Support Performance-Related Specification

The first role of the PBMD framework is to support a PRS system by finding the mathematical models of relationships between the volumetric AQCs and their performance before construction. During off-paving seasons, it would be helpful to conduct sensitivity analyses of the volumetric and/or mechanical AQCs on predicted performance so that the most sensitive AQCs and mathematical models can be determined. Full factorials of volumetric mix design scenarios, such as those explored in this study, need not be investigated. However, several alternative mix designs based on the experience and judgment of the contractor can be evaluated. It provides agencies and contractors guidance on how to adjust the AQCs in order to reach the performance targets, as well as a rational resource to calculate incentive or pay factor in the PRS system. Without the PBMD support in PRS, the uncertainty of performance predictions may result in a reluctant agreement between agency and contractor. If possible, they may select limited amounts of mechanical AQCs to improve the accuracy of performance predictions, but there may be significant reluctance to incorporate mechanical performance tests during production and construction.

**Figure 1:**
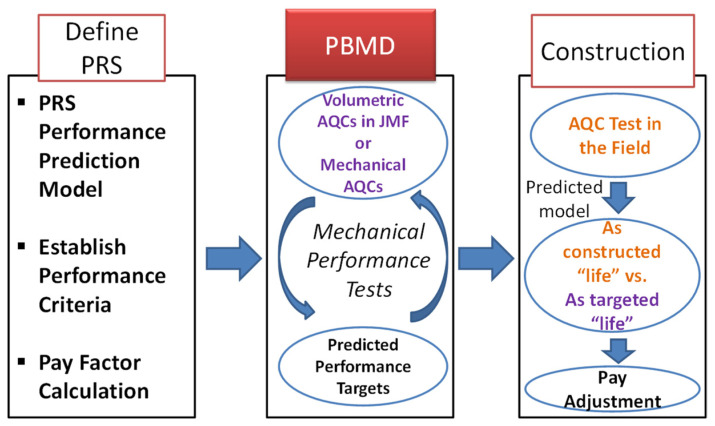
Role of performance-based mix design under the performance-related specification system.

Figure 1 demonstrates the role of the PBMD as a part of the PRS system in chronological order. At first, PRS performance models and the corresponding performance criteria and pay factor calculation method need to be defined by agency and contractor. Second, the mathematical models of relationships between the volumetric AQCs (i.e., design VMA and air void) in job mix formula or mechanical AQCs (i.e., performance index) measured by equipment such as an asphalt mixture performance tester (AMPT) and the predicted performance should be developed before construction. Not only does this provide guidance on how to adjust the asphalt mix design criteria to reach performance targets in the laboratory, but also on the acceptable variations of the volumetric or mechanical AQCs to control asphalt mixture’s quality during production. Lastly, the incentive or pay factor for the PRS system is calculated by comparing as-constructed predicted life with as-targeted predicted life based on the mathematical models in the PBMD phase.

#### 2.2.2. Performance-Based Mix Design to Support Long-Life Pavement

After understanding the means to adjust the volumetric mix design requirements for required performance targets, the second role of the PBMD framework is to develop three PBMD categories of the fatigue-preferred, rutting-preferred, and performance-balanced mix designs. Asphalt pavement structures consist multiple asphalt layers above the subgrade and base unbound layers. Structural analysis programs can consider the effect of the boundary conditions (i.e., pavement stress and strain responses due to traffic loading, temperature gradients along the pavement depth) of each asphalt layer on fatigue cracking and rutting performance. These programs allow asphalt mix designers to select the PBMD category of each asphalt layer that can address critical pavement distresses. To be specific, a binder-rich mix of the fatigue-preferred mix design could be selected to prevent the bottom-up cracking propagation with a lesser consideration of rutting issue in the bottom layer. In contrast, a less binder-rich and a strong aggregate skeleton for rutting-preferred mix design could be chosen to have rutting resistance, more friction, and less permeability in the surface layer. Lastly, based on the related research suggestions and the author’s team experience [17], polymer-modified binder mix with a strong aggregate skeleton of the performance-balanced mix design could be placed to prevent reflective bottom-up cracking and permanent deformation in the intermediate layer.

In this paper, the fatigue-preferred mix design is defined as the mix design that sacrifices but does not fail the rutting performance at the threshold value of rut depth, but largely improves the fatigue performance. In the same manner, the rutting-preferred mix design is defined as the mix design that shows the fatigue performance at the threshold value of cracking, but leads to a much better rutting performance. Lastly, the fatigue-rutting performance balance mix design is defined as the mix design that has the best trade-off between fatigue and rutting performance within possible performance targets that asphalt mix designers can develop.

**Figure 2:**
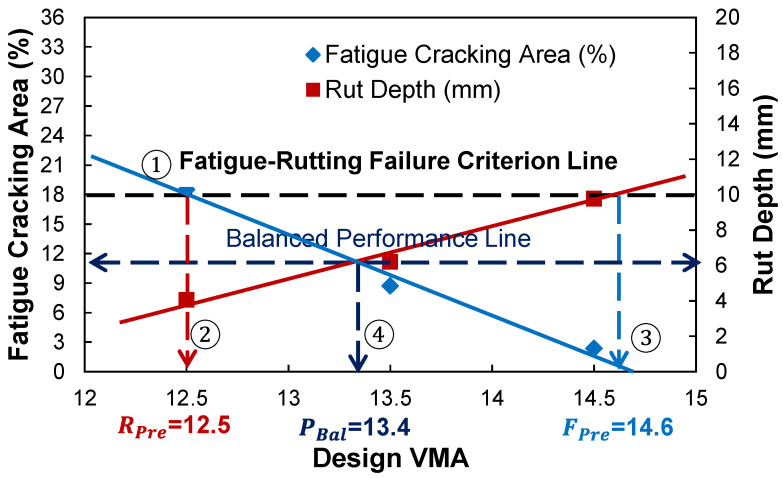
Analytical procedure to determine the performance targets and their volumetric AQC targets of three PBMD categories using the failure criterion and balanced performance lines.

Figure 2 describes the analytical procedure to determine the performance targets and their AQC values for the three mix design types using the fatigue and rutting criteria. The indication ①–④ represents the relation between intersection points of the criterion lines to the Design VMA. First, the relationship between the volumetric AQC and predicted fatigue and rutting performance are cross-plotted. The results of fatigue and rutting performance are separately expressed at the primary and secondary *y*-axis along with the same AQC values at the *x*-axis. By adjusting the scales of fatigue and rutting performance results at the primary and secondary *y*-axis, the fatigue cracking and rutting criteria can be placed at the same horizontal location so that a failure criterion line parallel to the *x*-axis can be drawn. This is because the normalized scale of fatigue and rutting performance by the failure criteria provides a balanced performance at the cross-point of the fatigue–AQC and rutting–AQC relationship lines. Second, the threshold value of the AQC for the rutting-preferred mix design is determined at the point where the failure criterion line and the fatigue cracking–AQC relationship line intercept. Third, in the same way, those of the fatigue-preferred mix design are determined at the cross-point between the failure criterion line and the rutting–AQC mathematical model. Fourth, the balanced performance targets of fatigue and rutting performance and their corresponding AQC values are found at the cross-point between the fatigue–AQC and rutting–AQC relationship lines.

#### 2.2.3. Suggestion of Performance-Based Mix Design Protocol

**Figure 3:**
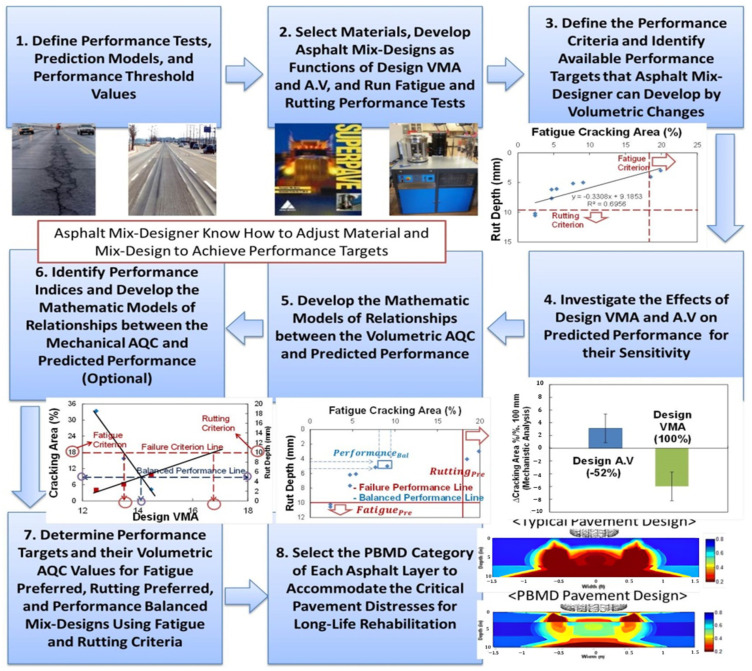
Suggested Performance-based mix design protocol.

In order to accomplish the aforementioned roles of the PBMD framework, this study suggests a protocol graphically summarized in Figure 3. The agency and contractor should first define which performance tests and analysis methods will be conducted to predict the fatigue and rutting performance of asphalt pavement. Then, the corresponding performance criteria need to be defined. After selecting asphalt materials, volumetric mix designs need to be developed to vary the design VMA and design air void that asphalt mix designers have under their control. After that, performance tests and analyses are conducted to predict the fatigue and rutting performance of the developed mix designs. In order to identify available performance targets that asphalt mix designer can develop through volumetric changes, the predicted performance of fatigue cracking and rutting are cross-plotted. Then, in order to determine which mix design parameter needs to be adjusted for performance targets by mix designers, a sensitivity analysis of individual volumetric design parameters on predicted performance is conducted. With the sensitivity results, the relationships between the sensitive volumetric AQC and its corresponding predicted performance need to be developed, and then the performance indices are identified by finding their correlations with the mechanistically predicted performance. After investigating the most sensitive fatigue and rutting performance index, the mathematical models of relationship between the determined mechanical AQCs and their corresponding predicted performance need to be optionally developed if the performance testing is available at the time of construction. The volumetric or mechanical AQC-predicted performance models can provide direction to mix designers toward the kinds of adjustment that should be made to achieve the required performance targets.

The performance targets and their AQC values of the fatigue-preferred, rutting-preferred, and performance-balanced mix designs need to be determined by following the analytical protocol explained in Figure 2. Finally, the PBMD category of each asphalt layer to accommodate the critical pavement distresses for long life can then be selected and the corresponding location within the pavement structure can then be determined. Complete bottom-up cracking propagation that results in a reconstruction of the entire asphalt pavement, along with severe rut depth that causes safety issues, such as hydroplaning and dangerous driving conditions, are considered the critical pavement distresses to be avoided.

## 3. Application of Suggested Performance-Based Mix Design Protocol

For the application of the suggested PBMD protocol, this study utilizes component materials from the recently reconstructed FHWA accelerated loading facility (ALF) and asphalt mixture performance tester (AMPT) equipment developed under the NCHRP Project 9–29, “Simple Performance Tester for Superpave Mix design.” These tools are combined with performance test methods, mechanistic models, and the three dimensional (3-D) pavement structural analysis program developed under the FHWA PRS project “Hot Mix Asphalt Performance-Related Specifications Based on Viscoelastoplastic Continuum Damage (VEPCD) Models” [34]. Using the ALF component materials, systematic experimental volumetric designs with three different VMA and air-void contents were developed using Superpave mix design principles and the Bailey method for aggregate gradation adjustments for VMA targets. After defining the performance threshold values from LVECD software output and developing the relationships between the AQCs and predicted performance, the performance targets and volumetric AQC values of three PBMD categories were determined. Finally, the appropriate PBMD categories of three asphalt layers were determined to avoid significant predicted distresses by comparing the performance results of PBMD pavement design with those of conventionally designed Superpave mixtures.

### 3.1. Define Performance Tests, Analysis Methods, and Performance Thresholds

Research under the FHWA PRS project has resulted in fatigue and rutting performance test methods and the corresponding mechanistic tests to feed models that can be performed on the AMPT. The fatigue cracking performance is determined by direct tension cyclic (DTC) testing and the simplified viscoelastic continuum damage (S-VECD) model, whereas rutting performance is characterized by triaxial stress sweep (TSS) testing and a viscoplastic shift model. The efficiency of these test methods and models allows the fatigue and rutting performance evaluation of asphalt mixture under a wide range of service conditions in four to five days. The LVECD program is the pavement structural performance prediction model that is compatible with the output from the AMPT. In order to efficiently reduce the computational time of the bottom-up and top-down cracking and rut depth predictions of asphalt pavement with seasonal temperature variation and traffic, a Fourier transform-based layered viscoelastic structural model has been adopted for the development of the LVECD program [35]. It was demonstrated that the LVECD program is capable of differentiating the top-down and bottom-up cracking pattern due to the viscoelastic material properties and boundary conditions of unbound layers under asphalt layers in a literature [15].

The performance threshold values used with LVECD results are based on the rulemaking program of national performance management measures as part of FHWA’s the Moving Ahead for Progress in the 21st Century Act [17]. In the rulemaking program, the pavement condition rating threshold values to classify three levels of performance (good, fair, and poor) are suggested as 5 and 10% for surface cracking area and 5 and 10 mm for rut depth.

#### 3.1.1. Testing Protocol

Uniaxial compressive dynamic modulus and the DTC fatigue tests were conducted to describe linear viscoelastic properties and viscoelastic damage characteristics of asphalt mixtures for S-VECD modeling in accordance with AASHTO TP79 and AASHTO TP107, respectively [25,36]. The dynamic modulus tests were completed at 5 °C, 22 °C, and 54.4 °C and at frequencies of 25, 10, 5, 1, 0.5, and 0.1 Hz. A test condition at 22 °C and at 0.01 Hz was added to better facilitate the construction of dynamic modulus master curves. Furthermore, the DTC fatigue tests were completed to fail at approximately 1000, 4000, 16,000, and 64,000 cycles at 18 °C and 10 Hz. Vertical deformations are measured using spring-loaded linear variable differential transformers (LVDTs) during dynamic modulus and DTC fatigue testing.

Triaxial repeated load permanent deformation (TRLPD) and triaxial stress sweep (TSS) tests were carried out to calibrate a reference permanent strain evolution curve (hereinafter called reference curve), reduced load time (time-temperature) shift factors, and vertical stress (time-stress) shift factors for viscoplastic shift modeling [24]. In addition to the rigorous mechanistic rutting analysis, incremental repeated load permanent deformation (iRLPD) tests were performed to construct minimum strain rate (MSR) master-curves used for rutting performance indices [8]. Three different deviatoric stresses are applied within one specimen to reduce the number of tests for the TSS and iRLPD tests. All permanent deformation tests were completed to apply cyclic haversine load followed by a rest period under confining pressure where the test specimens were enclosed with a latex membrane. The TRLPD data present a single loading block, including six hundred cycles with a single deviatoric stress and load time, while the TSS and iRLPD data demonstrate three and four loading blocks containing two hundred cycles of each loading block with different deviatoric stresses and a single load time in each. Detailed permanent deformation test conditions used in this study are provided in Table 2 [25,31,36]. Vertical deformations were measured using an AMPT actuator LVDT during iRLPD, TRLPD, and TSS testing and a steel ball was placed between the top platen and reaction frame which is different from the conventional flow number test protocol. The air-void target of the cylindrical specimens used for all fatigue and rutting tests was 7% +/− 0.5%.

#### 3.1.2. Fatigue Cracking Analysis Methods

Traditionally, bending beam fatigue tests have been conducted to determine classical fatigue relationships between several tensile strain levels at the bottom of the beam specimen and numbers of cycles to failure at three different temperatures. The main advantage of rigorous mechanistic S-VECD model coupled with the DTC fatigue tests allows the reliable prediction of fatigue relationships within one to two days. The S-VECD model is based on Schapery’s work of potential theory that uses internal state variable of damage S as following damage evaluation law [30].

(1) $$\dot{S}={\left(-\frac{\partial W^{R}}{\partial S}\right)}^{\alpha }$$

where,

S = internal state variable (damage),
$W^{R}$ = total dissipated pseudo strain energy, and
α = damage evolution rate.

**Figure 4:**
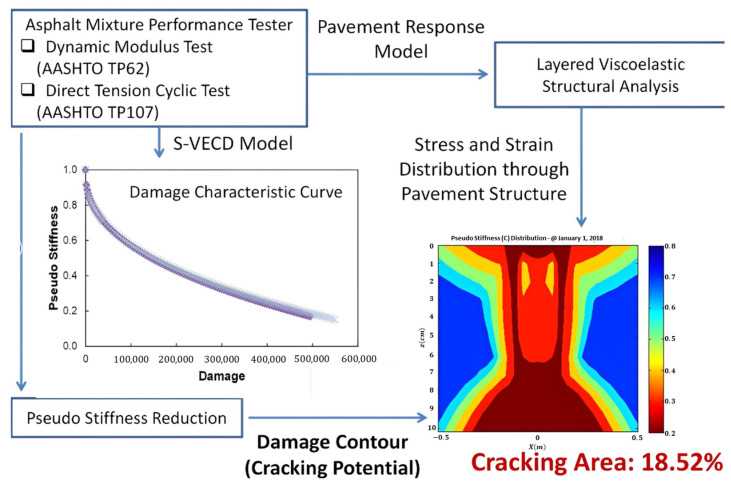
Mechanistic fatigue cracking area prediction method.

The main output from S-VECD is a damage characteristic curve that explains a relationship between pseudo-stiffness and a quantified damage state regardless of a strain level and temperature. The damage curve expresses the integrity of asphalt mixture as damage grows. The damage state at failure is determined at a peak phase angle during the DTC testing. Figure 4 demonstrates the mechanistic fatigue cracking analysis method used in this study to calculate the fatigue cracking damage percentage of the pavement cross-section cut through a vertical plane normal to the direction of traffic loading. The LVECD program can simulate the pseudo-stiffness (material’s integrity) accumulating damage reduction at all nodal points of the asphalt pavement depth and width. The pseudo-stiffness values at the failed damage conditions determined by observing the peak phase angle are considered as fatigue cracking initiation based on the S-VECD theory. Thus, the fatigue cracking area can be quantified by calculating a ratio of a number of nodal points that contain pseudo-stiffness values below the failure point relative to all nodal points in the designated area of the pavement structure. In this study, it is the pavement cross-section of ±0.5 m in width from the center of the loading tire through the whole asphalt thickness.

**Figure 5:**
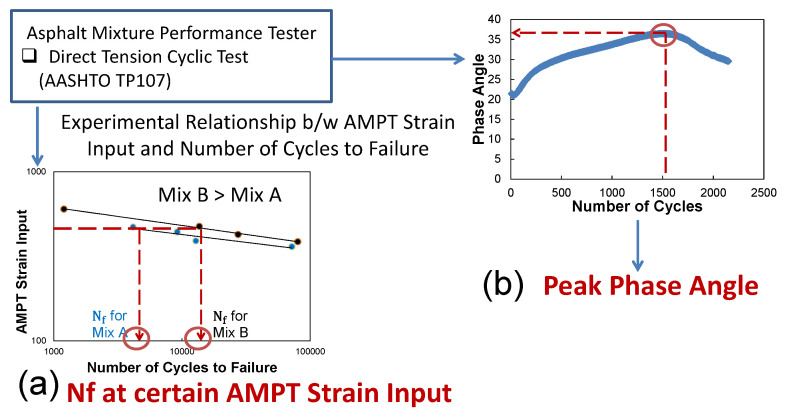
Fatigue cracking index method without mechanistic analysis.

Figure 5a,b describe fatigue performance indices that can be directly measured during the DTC tests without mechanistic analysis or post-processing of the data. In this study, two fatigue performance indices were evaluated: (1) the number of cycles to failure at a certain AMPT strain input value into the control software (this input may be different depending on a type of asphalt mixture) and (2) the average peak phase angle value at failure. These were investigated to identify the most sensitive fatigue index that has a strong correlation with mechanistic performance but requires significantly less time.

#### 3.1.3. Rutting Analysis Methods

**Figure 6:**
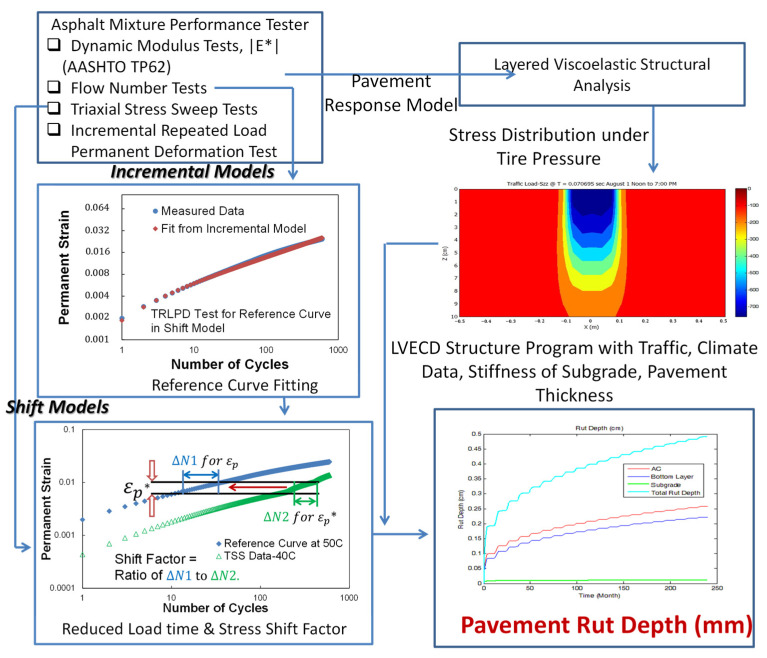
Mechanistic rut depth prediction method.

In mechanistic-empirical pavement design and analysis, permanent to resilient strain ratio models have been used to predict pavement rut depth as functions of a temperature, number of loading cycle, and resilient strain calculated from dynamic modulus tests. Since the strain ratio model does not take into account the effect of different deviatoric stress caused by various traffic loads, there is a need to develop a new viscoplastic strain model that can consider the effects of temperature, load time, and deviatoric stress. Recently, the viscoplastic shift model has been developed to predict permanent strain at any temperature, load time, and deviatoric stress using time-temperature and time-stress superposition principles. The shift model consists a reference permanent strain growth master curve, a so-called reference curve, a reduced load time (time-temperature superposition) shift factors, and vertical stress (time-stress superposition) shift factors. The reference curve is fitted using an incremental model as expressed in Equation (2). Choi and Kim found that the slopes of incremental permanent strains in log–log scale measured from each loading block of TRLPD and TSS tests are identical regardless of reduced load time and deviatoric stress [24]. This observation allows nine loading blocks of TSS tests measured at three different temperatures and at three different deviatoric stresses horizontally translating to the reference curve so that the reduced time shift factors and stress shift factors can be calculated as expressed in Equation (3). Each shift factor is calculated as a ratio of a number of cycles at the reference curve (∆N1) to a number of cycles at the TSS loading block that produces the identical amount of permanent strain (∆N2), as shown in Figure 6. The detailed incremental model and shift factor functional forms and analysis procedure can be found in an article by Choi and Kim [24].

(2) $$\varepsilon_{vp}=\frac{\varepsilon_{0}N_{ref}}{{\left(N_{I}+N_{ref}\right)}^{\beta }}$$

(3) $$N_{ref}=N\times 10^{a_{total}}\;a_{total}=a_{\xi_{p}}+a_{\sigma_{v}}\;a_{\xi_{p}}=p_{1}\log(\xi_{p})+p_{2},\;a_{\sigma_{v}}=d_{1}{\left(\frac{\sigma_{v}}{P_{a}}\right)}^{d_{2}}+d_{3}$$

where,

$\varepsilon_{vp}$ = viscoplastic (permanent) strain,
$\varepsilon_{0},N_{I},\beta$ = incremental model coefficients
$N_{ref}$ = number of cycles at the reference loading condition,
N = number of cycles at certain loading condition,
$a_{total}$ = total shift factor,
$a_{\xi_{p}}$ = reduced load time shift factor,
$a_{\sigma_{v}}$ = vertical stress shift factor,
$\xi_{p}$ = reduced load time,
$p_{1},p_{2}$ = regression parameters for reduced load time shift factor,
$\sigma_{v}$ = vertical stress,
$p_{a}$ = atmospheric pressure (i.e., 14.7 psi or 101.3 kPa), and
$d_{1},d_{2},d_{3}$ = regression parameters for reduced stress shift factor.

The output of the viscoplastic shift model are direct inputs to the LVECD program to predict pavement rut depth along with the EICM climate data, traffic, pavement thickness, and stiffness of unbound layers.

As was described above for fatigue, simple rutting performance indices that can be directly measured by AMPT without a mechanical structural analysis and data processing were also investigated. First, Azari and Mohseni [8] suggested constructing a minimum strain rate (MSR) master-curve that can calculate the “*b*” coefficient as expressed in Equation (4). Here, the minimum strain rates are measured at the last 50 cycles of each loading group in the iRLPD tests. The “*b*” coefficient is a slope of relationship between the MSR and TP temperature-and-pressure.

(4) $$MSR=a\times {\left(T\times P\right)}^{b}$$

where,

a,b = power function coefficients,
T = temperature (°C), and P = deviatoric stress (MPa).

**Figure 7:**
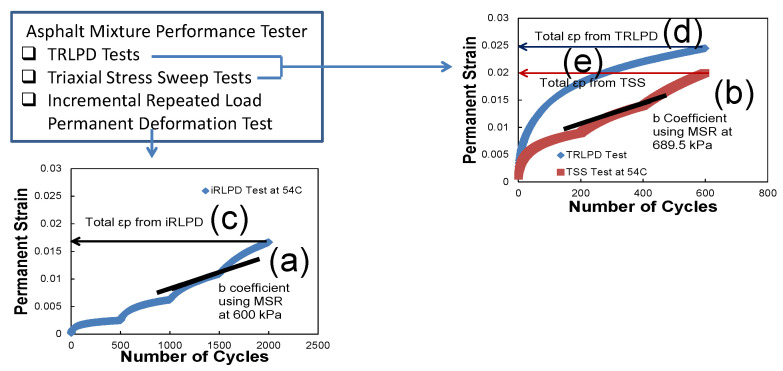
Rutting index method without mechanistic analysis: (a): b coefficient-MSR 600 kPa, (b): b coefficient-MSR at 689.5 kPa, total ερ from (c): iRLPD, (d): TLRPD; (e): TSS.

The “*b*” coefficient is suggested as a performance indicator to characterize rutting performance. It is calculated at the MSR value at 600 kPa by assuming the “*a*” coefficient is 0.001, as shown in Figure 7a. In the same way, the “*b*” coefficient can be determined from the TSS test by assuming that the “*a*” coefficient is 0.001 at the MSR value of 689.5 kPa, as shown in Figure 7b. The other simple rutting index is the total accumulated permanent strains measured from the iRLPD, TRLPD, and TSS tests as described in Figure 7c–e.

#### 3.1.4. Define Fatigue Cracking and Rut Depth Performance Thresholds

In this study, the rulemaking program of the national performance management measures as part of FHWA’s response to the Moving Ahead for Progress in the 21st Century Act (MAP-21) is used to define the fatigue and rutting performance threshold values. The proposed pavement condition rating thresholds are shown in Table 3. Based on the full-scale empirical mechanistic test [17] and the road and pavement expert’s suggestions, the extracted value were generated based on both asphalt pavement and asphalt pavement and jointed concrete pavement conditions under the AADT of 8000. Here, the failure criteria of rut depth (mm) and surface cracking area (%) of asphalt pavement are 10 mm and 10%, respectively.

The LVECD output for rutting performance is the calculated rut depth (mm) which has identical units in the rulemaking program. Therefore, the 10 mm of rut depth is determined as the rutting failure criterion. However, the current LVECD output for fatigue performance is the cracking propagation percentage of pavement cross-section. Therefore, the fatigue cracking damage percentage computed from the damage contour within the asphalt layer simulated by the LVECD program is determined as a failure criterion. A transfer function study is under development to convert the cracking damage area (%) of pavement cross-section to surface cracking area (%).

### 3.2. Asphalt Mix Designs with a Wide Variation in Design VMA and Air Void

One of the FHWA ALF test sections reconstructed in 2013 is a Superpave 12.5 mm nominal maximum aggregate size (NMAS) asphalt mixture with PG 64-22 binder and 22% of reclaimed asphalt pavement (RAP) by weight (20% recycled binder ratio). This mix was selected to base the suggested performance-based mix design framework. Using the same component materials (large stocks are available), new mix designs were developed to vary the asphalt mix design requirements for design VMA and design air void that are ordinarily under an asphalt mix designer’s control. Since the minimum volumetric criterion for a 12.5 mm NMAS mixture is 14% VMA, a slightly higher and lower design VMAs of 15% (as-constructed the ALF mix at design air void of 4%) and 14% were produced using the Bailey method. The Bailey method provides guidance to asphalt mix designers to determine different aggregate gradations that can predict design VMAs with aggregate packing concepts [29]. Loose unit weight and rodded unit weight tests are required to quantify the aggregate packing in accordance with AASHTO T 19 [37].

**Figure 8:**
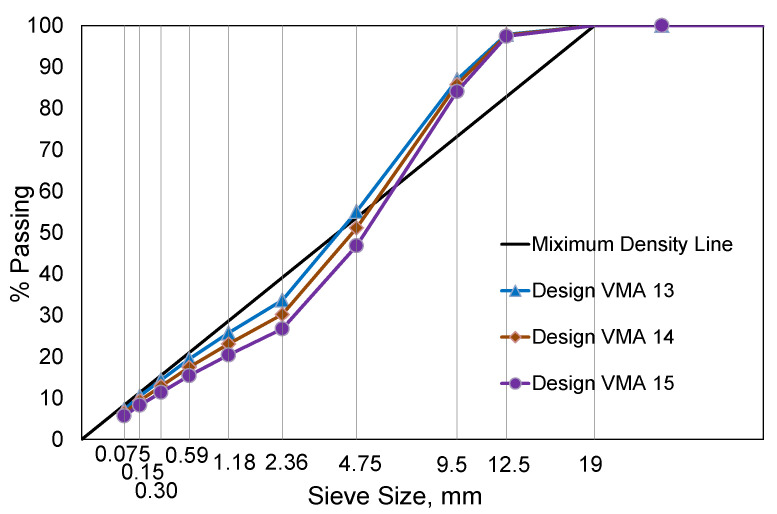
Three different aggregate gradations for the design VMAs.

After determining three different trial aggregate gradations, they were produced with two binder content alternatives at design air-void contents of 3 and 5% that try to meet the VFA requirement of 65% to 78% for design traffic ESALs of 0.3 to 3 million. The three different aggregate gradations and nine mix designs developed are summarized in Figure 8 and Table 4. Details can also be found in another article by Lee et al. [17]. The alphabetic identifiers from Mix-A to Mix-I shown in Table 4 are used to distinguish the performance results of individual mix designs.

### 3.3. Define the Performance Criteria and Identify Achievable Performance Targets

After completing the asphalt mix designs as functions of volumetric AQCs (design VMA and air void), the performance results of nine mix designs were investigated to identify achievable performance targets that asphalt mix designers can develop with reasonable volumetric changes. In this phase, the contrary relationship between fatigue and rutting performance may provide information on the best and worst performance targets the asphalt mix designers can achieve with a given set of materials. Furthermore, it may provide an indication to change aggregate gradation or modify virgin asphalt binder type to reach performance targets required by the agency and contractor. The LVECD program was utilized to predict the bottom-up and/or top-down fatigue cracking propagation as well as the rut depth of the nine mix designs. Seasonal temperature variations of the Washington D.C area were modeled with the Enhanced Integrated Climate Model (EICM) database and traffic was simulated with 4000 average annual daily truck traffic (AADTT) that provided about 4 million equivalent single axle loads (ESALs) in 5 years. The asphalt layer was 101.6 mm of (4 in) thick pavement. A single moving wheel load was simulated with a design velocity of 60 km/h, axle load of 45 kN (10 kips). The elastic moduli of for the subgrade were 70 MPa (10 ksi) and 150 MPa (22 ksi).

**Figure 9:**
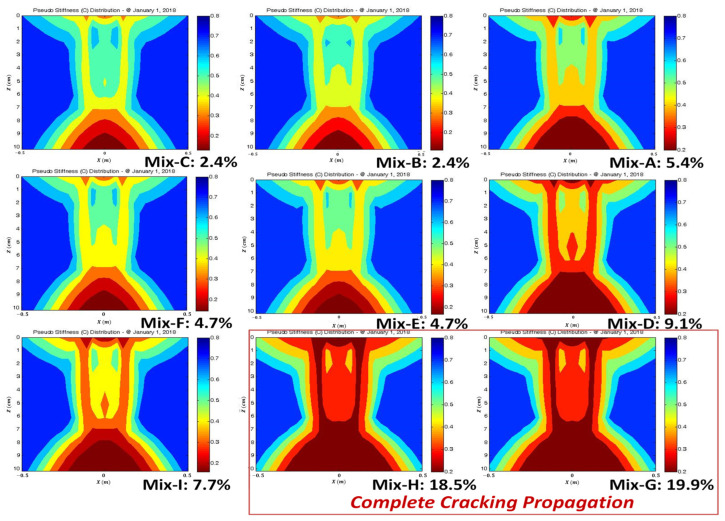
LVECD fatigue performance results: fatigue damaged area (%) results of nine mix designs at 101.6 mm (4 in) of thick pavement.

**Figure 10:**
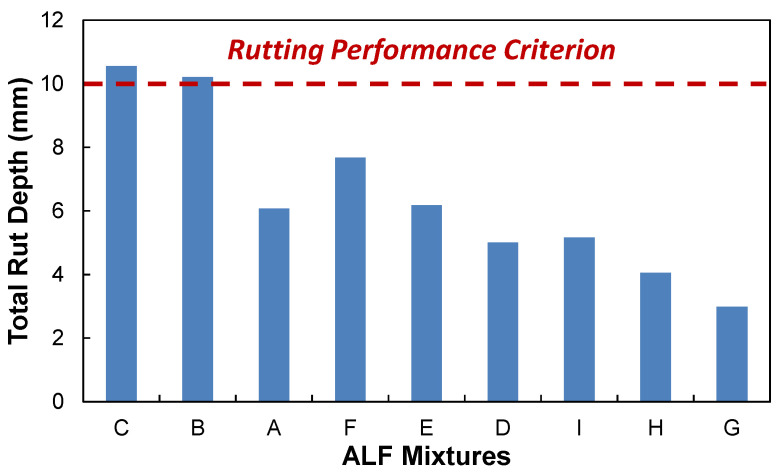
LVECD rutting performance results: rut depth (mm) of nine mix designs at 101.6 mm (4 in) of thick pavement.

Figure 9 and Figure 10 show the asphalt cross-section damaged area (%) and rut depth (mm) results modeled by the LVECD program. It is found that the results of the Mix-B, C, E, and F indicates that only bottom-up cracking propagations of 10 mm are observed when the fatigue damaged area was below 5%. Complete cracking damage was combined with bottom-up and top-down propagations for Mix-G and H when the fatigue damaged area was above 18%. Therefore, the fatigue cracking damaged area of 18% is selected as the fatigue cracking failure criterion from the LVECD program. In terms of rutting performance criterion, the accumulated asphalt layer rut depth of 10 mm is selected following the national rulemaking program.

**Figure 11:**
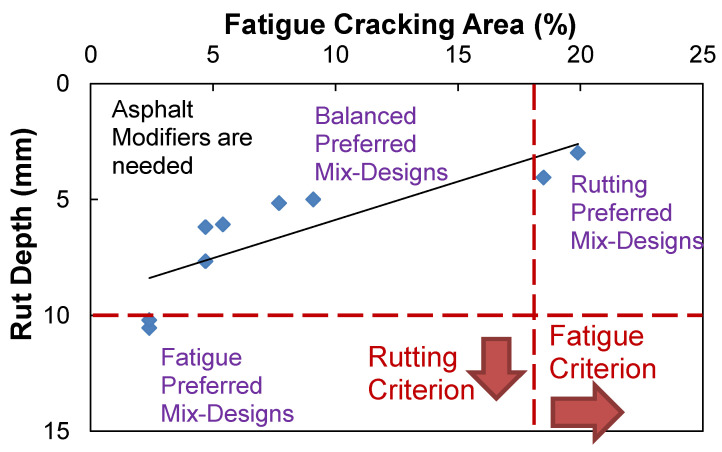
Fatigue and rutting performance cross-plot of the ALF mix designs.

Figure 11 presents the fatigue and rutting predictions cross-plotted that shows inverse relationships. The relationship can give information on the possible fatigue-preferred, rutting-preferred, and performance-balanced mix designs that are achievable by a mix designer with a given set of materials. Depending on circumstances, it may be concluded that the current asphalt mix design may need to modify the binder to obtain better engineering properties.

In the next section, the sensitivity analysis results for each volumetric AQC will be discussed; it provides guidance on the extent in which design VMA and air void need to be changed to reach certain performance targets.

### 3.4. Investigate the Effects of Design VMA and Design Air Void on Predicted Performance for Their Sensitivity

**Figure 12:**
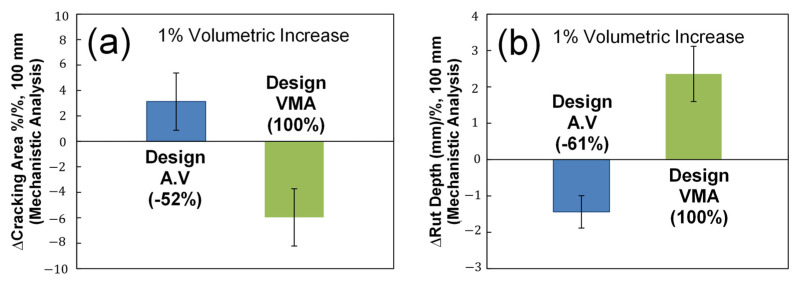
Sensitivity analysis results of individual volumetric AQCs on predicted mechanistic fatigue and rutting performance: (**a**) fatigue damage area (%) and (**b**) asphalt rut depth (mm).

A sensitivity analysis of design VMA and design air void was conducted to determine which volumetric parameter can be modified to meet required performance targets. The sensitivity was quantified by calculating the average slopes of linear relationships between the volumetric AQCs and predicted performance. The slope indicates how much the predicted fatigue and rutting performance can be changed with an increase of one percentage of the design VMA and air-void content. The sensitivity results of fatigue damage area (%) and asphalt rut depth (mm) are summarized in Figure 12. The most sensitive volumetric parameter is emphasized by the relative difference between the design VMA and air void, which is normalized to the greatest slope corresponding to 100%. Figure 12 illustrates that the design VMA is the more sensitive volumetric parameter that affects fatigue and rutting performance than design air void. It may be concluded that those consistent trends may imply that the deign VMA and air void can be used as volumetric AQCs that are related to fundamental engineering properties and their predicted performances in the PRS system.

### 3.5. Develop the Mathematical Models of Relationships between the Volumetric AQCs and Predicted Performance

**Figure 13:**
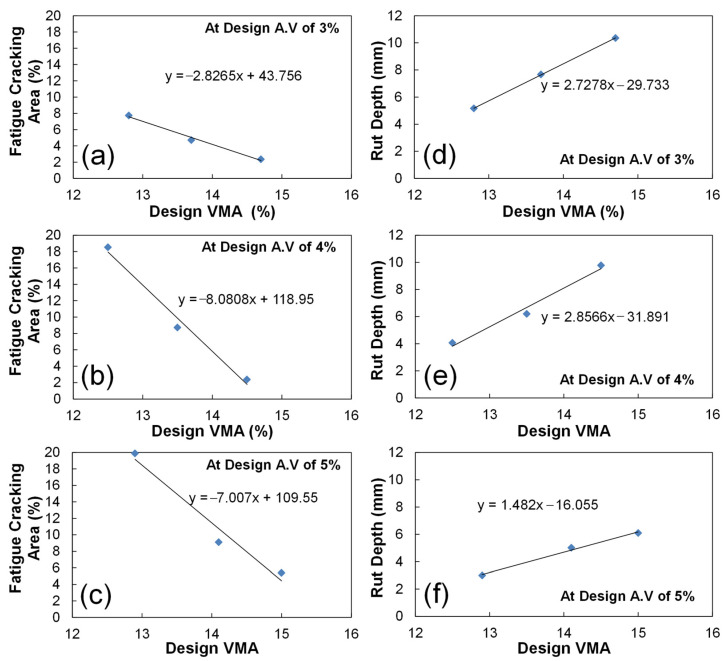
Development of mathematic relationships between the design VMA (%) and predicted performance (fatigue damage area (**a**–**c**), asphalt rut depth (**d**–**f**)) at design air voids of 3% (**a**,**d**), 4% (**b**,**e**), and 5% (**c**,**f**).

Based on the sensitivity analysis results, the mathematical models of relationships between the design VMA (the most sensitive volumetric AQC) and the predicted fatigue and rutting performance were developed at design air voids of 3, 4, and 5% as shown in Figure 13. Since those volumetric AQCs are interrelated, the design VMA-predicted performance relationship should be considered with the design air void. Figure 13 indicates that the relationships are very linear. Furthermore, it is observed that the design VMA is even more sensitive on predicted fatigue performance at design air voids of 4 and 5%, whereas it is more sensitive on predicted rutting performance at design air voids of 3 and 4%. This implies the aggregate gradation plays a more important role on the fatigue performance with low binder content and rutting performance with high binder content.

### 3.6. Identify Performance Indices (Mechanical AQCs) and Their Performance Threshold Values for Quality Assurance in PRS System

If asphalt mixture performance testers (AMPTs) are available to conduct the quality assurance during construction, the performance indices (mechanical AQCs in PRS system) may be used as a more accurate way to predict pavement performance. Otherwise, predictive relationships that are under development would have to be used and would have some unavoidable error. In this section, the relationship between proposed performance indices shown in previous sections that can be obtained by a faster or simpler interpretation of the fatigue and rutting mechanical tests and the already-completed mechanistic analysis results were quantified to find the indices that could more efficiently predict fatigue and rutting performance.

**Figure 14:**
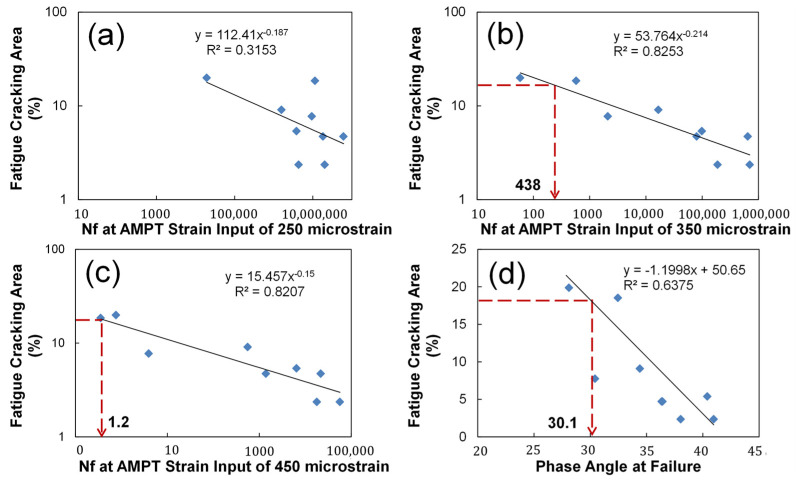
Identification of the optimal fatigue cracking index and its performance threshold value: (**a**) 250 micro-strain; (**b**) 350 micro-strain; (**c**) 450 micro-strain; (**d**) Phase angle at failure.

The number of cycles to failure at a certain AMPT software strain input and averaged phase angle value at failure were investigated to determine the best fatigue index as shown in Figure 14a–d. Here, the AMPT software strain input is the value the user provides in the software control prompt for the averaged on-specimen strain target value during the DTC fatigue testing. The advantage of this index is not having to run any S-VECD analytical structural simulations, only a quick interpretation of the physical test. It is found that the number of cycles to failure (fatigue life) at the AMPT software strain input of 250 microstrain has a poor correlation with the mechanistic fatigue cracking performance with an R-square of 0.32, while the fatigue life at the AMPT inputs of 350 and 450 microstrains have stronger correlations with the mechanistic cracking area with an R-square of 0.83 and 0.82, respectively, as shown in Figure 14a–c. Since a failure criterion of 1.2 loading cycles is impractical at the AMPT strain input of 450 microstrain, the fatigue life at the AMPT strain input of 350 microstrain is selected as a fatigue performance index candidate. For example, with this particular set of materials, using a 350 microstrain input to the control software, the cycles to failure was about 438 cycles, and the test would only need to be run for 44 s, less than a minute. In addition, Figure 14d illustrates a good relationship between the averaged phase angle at failure and predicted fatigue performance by an R-square of 0.64.

**Figure 15:**
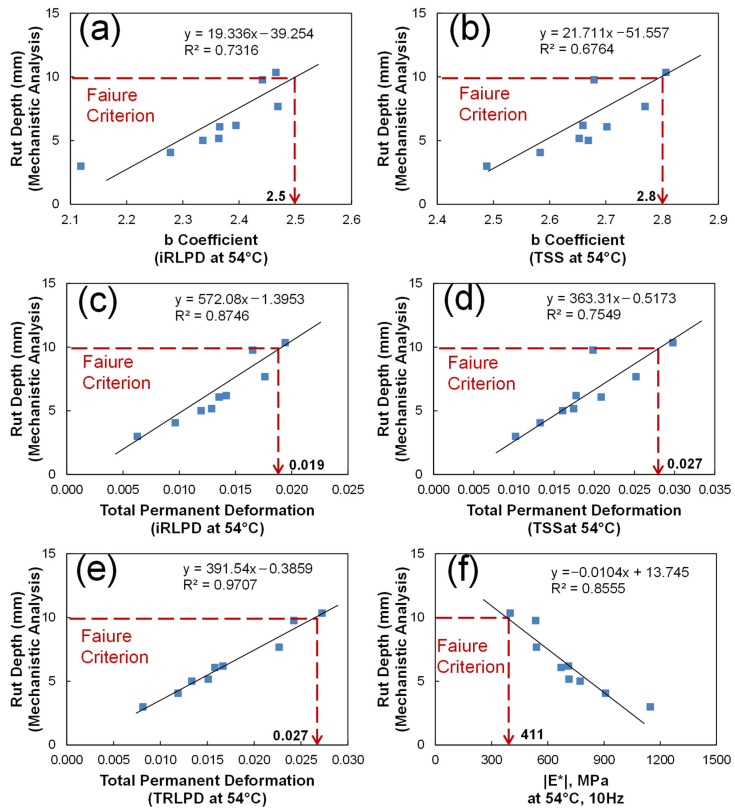
Identification of the optimal rutting index and its performance threshold values: b Coefficient (**a**) iRLPD, (**b**) TSS; Total permanent deformation at: (**c**) iRLPD, (**d**) TSS, (**e**) TRLPD; $\left|E^{*}\right|$: (**f**).

In the same way to identify the optimal fatigue index, an efficient rutting index is investigated. Figure 15a,b show the correlation of “b coefficient”, the slope of minimum strain rate master-curve, from iRLPD and TSS tests at 54 °C with the mechanistic rut depth having an R-square of 0.73 and 0.67, respectively. Figure 15c–e present the linear relationship between the total accumulated permanent deformation (strain) from iRLPD, TSS, and TRLPD tests at 54 °C and the mechanistic rut depth. Figure 15f illustrates the negative correlation of the dynamic modulus at 54 °C and 10 Hz with the mechanistic rut depth by an R-square of 0.86. It is concluded that the total accumulated permanent strain is an optimal rutting index that can efficiently predict mechanistic rutting performance for these materials studied with a threshold value of 0.027.

**Figure 16:**
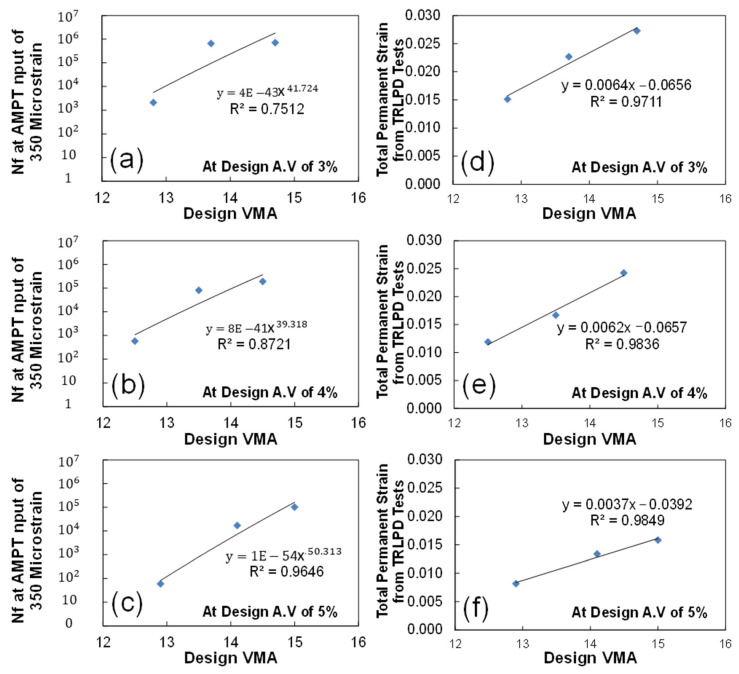
Development of the mathematical models on the relationship between the performance index and predicted performance (AMPT (**a**–**c**); TRLPD (**d**–**f**)) at design air voids of 3% (**a**,**d**), 4% (**b**,**e**), and 5% (**c**,**f**).

Relationships between the efficient performance indices and predicted mechanistic performance were compared in the same way as the volumetric AQC-predicted performance linear models as expressed in Figure 16. The results show that the model form of fatigue index is a power function, whereas that of rutting index is a linear function. The rutting index has a stronger relationship with the mechanistic performance rather than the fatigue index. Similar to the sensitivity of the design VMA on the mechanistically predicted performance, the design VMA is also more sensitive on the fatigue index at the design air voids of 4 and 5%, even though it is more sensitive on the rutting index at 3 and 4%. These volumetric or mechanical AQCs-predicted mechanistic performance mathematical models provide guidance on the extent in which the design VMA or performance indices need to be adjusted in order to reach the specific performance targets at the time of construction during production.

### 3.7. Determine Performance Targets and Their Volumetric AQC Values for Fatigue-Preferred, Rutting-Preferred, and Performance-Balanced Mix Designs

In order to develop the fatigue-preferred, rutting-preferred, performance-balanced mix designs, their performance targets need to be defined. In the phase for defining performance thresholds in the PBMD framework, the performance targets of fatigue and rutting-preferred mix designs for the LVECD output are determined, but the balanced performance target is not yet determined. Since the volumetric change of asphalt mixtures results in a reverse trend between fatigue and rutting performance, as shown in Figure 11, asphalt mix design that has the rutting performance at the threshold value of 10 mm may provide the fatigue-preferred mix design. In the same way, the fatigue performance at the threshold value of 18% may produce a rutting-preferred mix design. Finally, the performance-balanced mix design may need to produce the best balanced performance of fatigue and rutting within the available performance targets that mix designers can develop.

**Figure 17:**
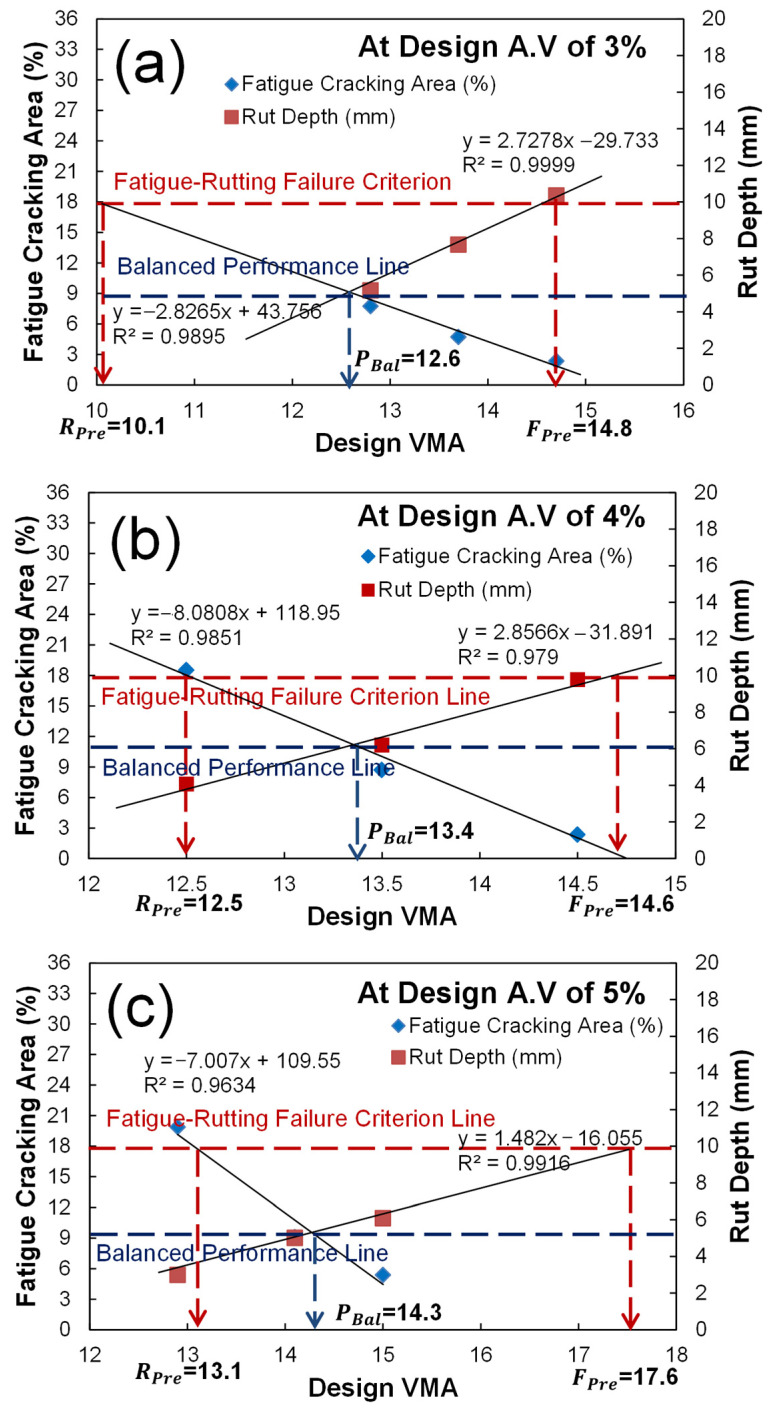
Results of the performance criteria and volumetric AQC targets of fatigue-preferred, rutting-preferred, and performance-balanced mix designs: (**a**) air void 3%; (**b**) air void 4%; (**c**) air void 5%.

Figure 17 shows the way to determine the performance criteria and their volumetric AQC targets of three PBMD categories using the failure criterion and balanced performance lines. In order to determine these lines, the two relationships developed in Figure 13 need to be cross-plotted together. After normalizing the fatigue and rutting performance results in primary and secondary *y*-axis by the their threshold values of 18% and 10 mm, the failure criterion line connected between the fatigue cracking of 18% and rut depth criteria of 10 mm, and the balanced performance line parallel to the *x*-axis at the cross-point where the two relations intercept, could be drawn as shown in Figure 17.

Table 5 summarizes the results of fatigue and rutting performance targets and their corresponding design VMA values for the three PBMDs. The range of performance targets and AQC values are measured at design air voids of 3, 4, and 5%.The determined performance targets and corresponding volumetric AQC of fatigue-preferred mix design are fatigue cracking of 0 to 1.9%, rut depth of 10 mm, and design VMA of 14.8 to 17.6%, whereas those of rutting-preferred mix design are fatigue cracking of 18%, rut depth of 0 to 3.8 mm, and design VMA of 10.1 to 13.1%. In addition, those of performance-balanced mix design are fatigue cracking of 8.1 to 10.7%, rut depth of 4.6 to 6.4 mm, and design VMA of 12.6 to 14.3%. It is interesting to observe that the performance-based mix design has the best balanced performance at a design air void of 3%.

### 3.8. Select the PBMD Category of Each Asphalt Layer to Accommodate Critical Pavement Distresses for Long-Life Rehabilitation

**Figure 18:**
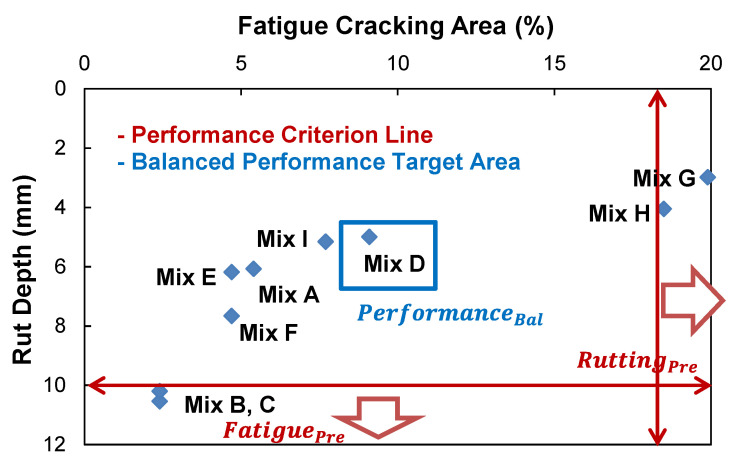
Selected PBMD mix designs based on performance levels for structural simulations.

The final role in the PBMD framework is to select the category of each asphalt layer that can accommodate the critical distresses for longer life through the LVECD program. The inputs of each asphalt layer used in the LVECD program are the fundamental material properties that mechanistically predict fatigue and rutting performance under the in-service conditions. In order to determine those fundamental material properties of three PBMD categories, the determined performance targets of three PBMDs are expressed in the performance cross-plot as shown in Figure 18. Mix-C that slightly exceeds the rut depth of 10 mm and provides a fatigue cracking damage area of 2.4% is selected as the fatigue-preferred mix design. In the same way, the Mix-G is selected as the rutting-preferred mix design. Finally, Mix-D contained in the balanced performance target area is selected as the performance-balanced mix design for the pavement structural analysis example.

A performance-optimized PBMD structural configuration is compared with full depth Superpave for a deep perpetual pavement scenario. The total asphalt thickness is 254 mm (10 in), consisting three layers. The LVECD program was run to evaluate fatigue and rutting performance in the Washington D.C area with a traffic loading of 8000 AADTT for 44 million ESALS over 30 years.

A Superpave structural design was analyzed to compare to a performance optimized structure. The Mix-E, having the minimum design VMA of 14, is selected for the 76.2 mm (3 in) thick surface layer. Mix-D, which is stiffer than Mix-E, is selected for the 101.6 mm (4 in) intermediate layer. Lastly, the 76.2 mm (3 in) thick bottom layer is chosen as Mix-G, which is stiffer than the Mix-D.

PBMD structural configuration should reduce complete bottom-up cracking propagation and resist rutting so that only surface milling and resurfacing can maintain the overall pavement structure in a sound condition over 50 years. Following the perpetual pavement concept, the fatigue-preferred mix design, Mix C, is selected for the 76.2 mm (3 in) thick bottom layer of the pavement. The performance-balanced mix design is selected to delay the bottom-up cracking propagation and have the rutting resistance for the 101.6 mm (4 in) intermediate layer. Finally, the rutting-preferred mix design is selected to have a rutting resistance and friction for the 76.2 mm (3 in) thick surface layer in accordance with the perpetual pavement concept.

**Figure 19:**
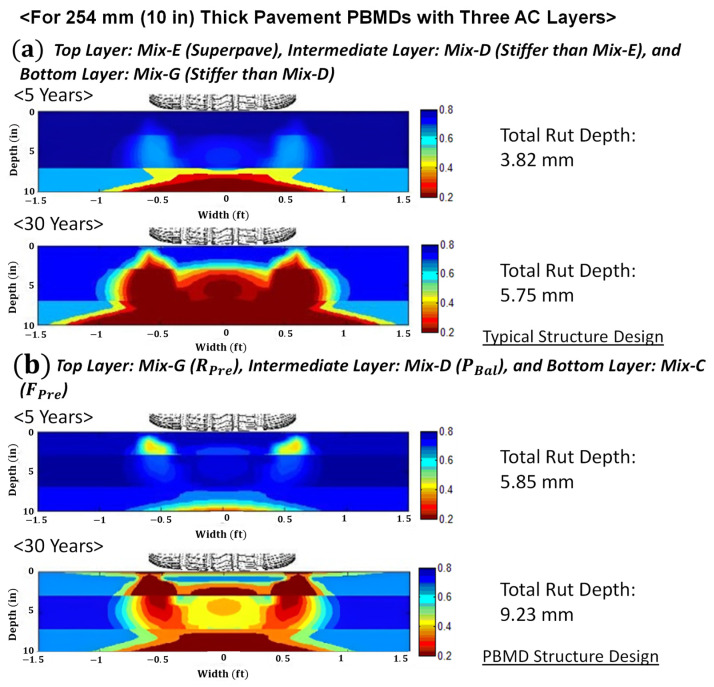
Comparison of full-depth Superpave with the PBMD structural configuration.

In Figure 19, the simulation results from the LVECE program show that the PBMD structural configuration can reduce the bottom-up cracking propagation without exceeding the rutting performance criterion for a long-life pavement management system. The simulation results also imply that the asphalt mixture in the surface layer may need to have a higher performance asphalt binder with modifiers to better resist top-down fatigue performance.

## 4. Conclusions

The proposed PBMD framework presents a comprehensive approach for supporting a PRS system and long-life pavements. The framework involves the development of various components, such as performance tests, prediction models, and threshold values. By doing so, it provides a systematic and standardized way of assessing the performance of asphalt pavements:

▪ One of the key features of the proposed framework is the identification of achievable performance levels through volumetric changes. Mix designers can develop asphalt mix designs that vary design voids VMA and design air void, which are factors that they have control over. By varying these design parameters, designers can achieve different performance levels, and the framework provides a way to identify these levels.
▪ Another critical aspect of the framework is the investigation of the effect of volumetric requirements on predicted performance for their sensitivity. The sensitivity analysis found that the design VMA is the most sensitive volumetric AQC that mix designers need to control for required performance targets. Moreover, mathematical models were developed to establish very linear relationships between the volumetric AQC of design VMA and the predicted performance to support the PRS.
▪ Efficient performance indices were also developed to facilitate the PRS system. The indices for fatigue and rutting were the number of cycles to failure at the AMPT software strain input of 350 microstrain and total accumulated permanent strain measured from the TRLPD tests. By using these indices, it is possible to evaluate the performance of pavements and determine if they meet the required criteria.
▪ Furthermore, the proposed framework determined the performance targets and their AQC values of three PBMD types using predicted performance criteria. For the fatigue-preferred mix design, the performance targets were a fatigue cracking area of 0 to 1.9% and a rut depth of 10 mm from a design VMA of 14.8 to 17.6%. The rutting-preferred mix design had a fatigue cracking area of 18% and a rut depth of 0 to 3.8 mm from design VMA as low as 10.1 to 13.1%. Additionally, the performance-balanced mix design criteria were a fatigue cracking area of 8.1 to 10.7% and a rut depth of 4.6 to 6.4 mm from design VMA of 12.6 to 14.3%. The performance-based mix design had the best-balanced performance at a design air void of 3%.
▪ Finally, the proposed PBMD pavement design with the fatigue-preferred mix design placed in the bottom layer, performance-balanced mix design in the intermediate layer, and rutting-preferred mix design in the surface can reduce the complete bottom-up cracking propagation without exceeding the rutting performance criteria. Simulation results from the LVECD structural analysis software verified the effectiveness of this design in achieving long-life pavements.
▪ Considering the limitation of this research, the need for further validation of the proposed framework through field testing and a verification of its effectiveness in different climatic and traffic conditions should be greatly considered. Additionally, future work could focus on incorporating environmental and economic factors into the framework to provide a more comprehensive approach to pavement design and maintenance.
▪ Overall, the proposed PBMD framework provides a robust and structured approach to support a PRS system and long-life pavements, enabling efficient and effective design and maintenance of asphalt pavements.

## Figures and Tables

**Table 1:** Comparison of asphalt mix designs currently used in the U.S.

Component	Hveem Mix Design	Marshall Mix Design	Superpave Mix Design
Aggregate selection method [31,32]	LA abrasion, sulfate soundness, polishing, crushed face count, flat and elongated particle count	LA abrasion, sulfate soundness, polishing, crushed face count, flat and elongated particle count	Angularity for internal friction, flat and elongated particles for aggregate breakage, clay content for adhesive bond, toughness by LA abrasion test, soundness by sodium or magnesium sulfate test, gradation control points
Asphalt binder selection method [31,33]	Asphalt cement grade for type and geographical location	Asphalt cement grade for type and geographical location	Performance grade by LTPP Bind software and AASHTO Superpave program, for original binder, flash point, rotational viscosity, and dynamic shear rheometer for rolling thin-film oven-aged binder, mass loss and dynamic shear rheometer, for pressure-aging vessel-aged binder, dynamic shear rheometer and bending beam rheometer
Compaction method [7,31]	Kneading	Drop Hammer	Gyratory
Volumetric mix design requirement [7,31]	Hveem stability and air void	Marshall stability, flow, air void, VMA	Air void, VMA, VFA, dust-to-binder ratio

**Table 2:** TRLPD, TSS, and iRLPD test conditions.

Test Type	Viscoplastic Shift Modeling		MSR Master-Curve
	TRLPD	TSS	iRLPD
Testing temperature (°C)	54	54, 40, 20	54
Confine pressure (kPa)	68.95 (10 psi)		
Pulse time (s)	0.4	0.4	0.1
Rest period	10	10 at 54 °C, 1.6 at 40 °C, 20 °C	0.9
Deviatoric Stress (kPa)	689.5 (100 psi)	482.6 (70 psi), 689.5 (100 psi), 896.3 (130 psi)	200 (29 psi), 400 (58 psi), 600 (87 psi), 800 (116 psi)
Number of cycles for each loading block	600	200	500
Testing time (min)	104	104 at 54 °C, 20 at 40 °C and 20 °C	35

**Table 3:** Proposed pavement condition rating thresholds from rulemaking.

Surface Pavement Type	Metric	Metric Range	Rating
Asphalt pavement	Rutting	<5 mm (0.2 in)	Good
		5 mm (0.2 in) to 10 mm (0.4 in)	Fair
		>10 mm (0.4 in)	Poor
Asphalt pavement and jointed concrete pavement	Surface cracking percentage	<5%	Good
		5 to 10%	Fair
		>10%	Poor

**Table 4:** Volumetric mix designs for three different design VMAs and design air-void contents.

(%)	Aggregate Gradation 1 (VMA 15 Target)			Aggregate Gradation 2 (VMA 14 Target)			Aggregate Gradation 3 (VMA 13 Target)		
Design VMA by volume	15	14.5	14.7	14.1	13.5	13.7	12.9	12.5	12.8
Design AV by volume	5.3	3.8	3	4.9	3.7	2.9	5.1	3.9	3.1
Binder content by weight *	4.2	4.5	4.9	3.8	4.1	4.4	3.2	3.6	3.9
Gmm	2.769	2.754	2.735	2.775	2.760	2.746	2.803	2.783	2.769
VFA by volume	64.7	73.8	79.6	65.2	72.6	78.7	60.5	68.8	75.8
Performance specimen AV	7 Mix-A	7 Mix-B	7 Mix-C	7 Mix-D	7 Mix-E	7 Mix-F	7 Mix-G	7 Mix-H	7 Mix-I

* the specific gravity of the aggregate is very high at nearly 3.00, and thus, if it were a typical stone near 2.70, then the binder contents by weight would be approximately 0.5% higher.

**Table 5:** Results of the performance targets and their volumetric AQC values of the three PBMDs at design air voids of 3, 4, and 5%.

PBMD		Fatigue-Preferred Mix Design			Rutting-Preferred Mix Design			Performance-Balanced Mix Design		
Design air void (%)		3	4	5	3	4	5	3	4	5
Performance targets	Cracking (%)	1.9	0	0	18	18	18	8.1	10.7	9.3
	rut depth (mm)	10	10	10	0	3.8	3.4	4.6	6.4	5.1
Design VMA (%)		14.8	14.6	17.6	10.1	12.5	13.1	12.6	13.4	14.3

## Data Availability

Data will be provided upon request.
